# Tomato AUXIN RESPONSE FACTOR 5 regulates fruit set and development via the mediation of auxin and gibberellin signaling

**DOI:** 10.1038/s41598-018-21315-y

**Published:** 2018-02-14

**Authors:** Songyu Liu, Youwei Zhang, Qiushuo Feng, Li Qin, Changtian Pan, Anthony Tumbeh Lamin-Samu, Gang Lu

**Affiliations:** 1 Key Laboratory of Horticultural Plant Growth, Development and Quality Improvement, Ministry of Agricultural, Department of Horticulture, Zhejiang University, Hangzhou, 310058 China; 2 Zhejiang Provincial Key Laboratory of Horticultural Plant Integrative Biology, Zhejiang University, Hangzhou, 310058 China

## Abstract

Auxin response factors (ARFs) encode transcriptional factors that function in the regulation of plant development processes. A tomato ARF gene, *SlARF5*, was observed to be expressed at high levels in emasculated ovaries but maintained low expression levels in pollinated ovaries. The *ami*RNA *SlARF5* lines exhibited ovary growth and formed seedless fruits following emasculation. These parthenocarpic fruits developed fewer locular tissues, and the fruit size and weight were decreased in transgenic lines compared to those of wild-type fruits. Gene expression analysis demonstrated that several genes involved in the auxin-signaling pathway were downregulated, whereas some genes involved in the gibberellin-signaling pathway were enhanced by the decreased *SlARF5* mRNA levels in transgenic plants, indicating that SlARF5 may play an important role in regulating both the auxin- and gibberellin-signaling pathways during fruit set and development.

## Introduction

The plant hormone auxin, indole-3-acetic acid (IAA), plays an important role in various aspects of plant development, such as cell extension, division, and differentiation, as well as in organ and tissue development, and tropism. Auxin response factors (ARFs) bind specifically to a TGTCTC motif found in auxin-responsive promoter elements (AuxREs) and mediate auxin responses. A typical ARF protein contains three parts: an N-terminal DNA-binding domain (DBD), a carboxyl-terminal dimerization domain (CTD) that is similar to motifs III and IV of Aux/IAA proteins, and a middle region (MR) that may activate or repress the expression of early auxin response genes, including small auxin up RNA (SAUR), Gretchen Hagen-3 (GH3) and lateral organ boundaries-domain (LBD)^1^.

Among the components of the auxin signal transduction pathway, ARF proteins participate in transcriptional regulation of a variety of biological processes related to plant growth and development. Studies of *arf* mutants have indicated that different ARFs possess diverse functions, which is due to the differences in temporal and spatial expression and affinities with promoters of target genes. Recently, functional studies have demonstrated that ARF genes play an essential role in signal transduction during plant organ development. In *Arabidopsis thaliana*, ARF2 regulates leaf senescence^2^, root formation and flower organ senescence^3^. Moreover, heterologous expression of mango *MiARF2* in *Arabidopsis* inhibits root and hypocotyl growth^4^. ARF3 plays an important role in lateral root development of *Arabidopsis* and regulates epidermal cells and trichome formation in tomato^5,6^. S1ARF4 is involved in hypocotyl development, cotyledon growth and fruit pericarp and sugar metabolism during tomato fruit development^7,8^. Inhibition of *ARF2*, *ARF3*, and *ARF4* expression by some tasiRNAs may release the repression of Arabidopsis lateral root growth^9^. Arabidopsis ARF10 and ARF16 have essential roles in the process of root cap development, and the auxin signal cannot bypass them to initiate columella cell production^10^. Furthermore, ARF7 and ARF19 may regulate Arabidopsis LR formation initiation by interacting with IAA14^11^.

Several ARFs also play an essential role in reproductive development. Tomato SlARF2 is involved in the regulatory mechanism of the fruit ripening process^12^. In *Arabidopsis*, ARF6 and ARF8 promote jasmonic acid production and flower maturation^13^ and influence the developing floral organs^14^. Downregulation of *SlARF6* and *SlARF8* in transgenic tomato plants by the overexpression of *MIR167a* may lead to floral development defects and female sterility^15^. Furthermore, ARF8 is a negative regulator of fruit initiation and development in *Arabidopsis*, tomato and eggplant^16,17^. Similarly, SlARF7 functions as a negative regulator of fruit set until pollination and fertilization have occurred^18^. Models have been proposed for the effects of ARF8^19^, SlARF7 and SlARF9^18,20^ on plant fruit development through the interactions between the auxin- and GA-signaling pathways during tomato fruit initiation and development.

Tomato–a fleshy climacteric fruit–is a model plant for studying fruit development and ripening^21^. In general, tomato fruit development is divided into four major phases: fruit setting, cell division, cell expansion, and a fruit ripening period. Phytohormones have been demonstrated to participate in the regulation of every aspect of fruit development; however, each hormone plays a specific role in the fruit set, expansion, and ripening stages. Biochemical analyses have identified two peaks in auxin levels during tomato fruit development: the first at 8 d after pollination at the end of active cell division, and the other at 30 d after pollination^22^, corresponding to cell expansion (stage III) and the fruit maturation stage (stage IV), thereby suggesting that auxin has an important role in promoting fruit cell expansion^22–24^ and initiating and enhancing climacteric ripening^25^. On the other hand, the levels of endogenous GAs peak in two stages: first at 8 d after flowering and then approximately 15 d before ripening^22,26^, which corresponds to cell division (stage II) and the cell expansion stage (stage III) of fruit development, thereby indicating that GA is an important factor in fruit cell cycle and expansion^27,28^. GA activity regulates the expression of cell division and the expansion genes in tomato^29^. Furthermore, previous studies have demonstrated that the expansion of tomato locular cells coinciding with the expression of genes encoding for water flow, organic acid synthesis, sugar storage, and photosynthesis is regulated by auxin and GA^30^. SlIAA17 in the auxin-signaling pathway may affect fruit cell size in tomato^31^. Additionally, Matsuo *et al*.^32^ suggested that cytokinins regulate fruit development by modulation of auxin biosynthesis and/or polar auxin transport. Certain cell cycle genes, such as cyclin-dependent kinases (CDKs)^33^ and cell cycle-associated protein kinase WEE1^34^, affect tomato fruit cell division. Meanwhile, certain other genes, including *TAGL1* (SHATTERPROOF)^35^, MYB factors^36^, and *SlPPC2* (phosphoenolpyruvate carboxylase)^37^, may regulate fruit cell expansion in tomato.

In a previous study, we identified 21 *SlARFs* in the tomato genome; their structures, chromosome locations, and phylogeny were predicted^38^. However, to date, their functions in fruit development have not been studied (beyond SlARF6, SlARF7, SlARF8 and SlARF9). Our data indicated that certain other ARFs also exhibited preferential expression in young ovaries and their mRNA levels were significantly altered during fruit development. Their roles in fruit development remain unclear. To explore the function of SlARF5 during fruit development, *SlARF5*-silenced tomato plants were generated by the *ami*RNA method. *SlARF5 ami*RNA lines form seedless fruits without pollination after emasculation. These parthenocarpic fruits developed fewer locular tissues with reduced fruit size and weight compared to those of wild-type plants. Downregulation of *SlARF5* resulted in reduced expression of auxin-related genes and increased the expression levels of certain GA-signaling pathway genes. According to these data, we hypothesized that SlARF5 may participate in the regulation of early fruit set and development by mediating the crosstalk between IAA and GA hormones during tomato fruit development.

## Results

### Expression pattern of *SlARF5* in tomato

**Figure 1:**
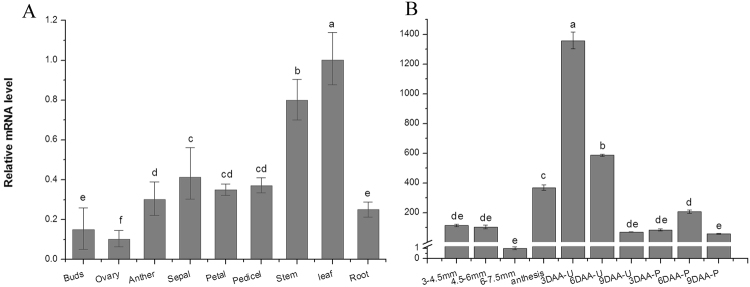
Real-time PCR analysis of relative gene expression of *SlARF5* mRNA in tomato. (**A**) *SlARF5* transcripts in different organs and tissues. (**B**) Expression levels of *SlARF5* at different stages during flower and fruit development. Flower bud samples were at lengths of 3.0–4.5 mm, 4.5–6.0 mm, and 6.0–7.5 mm. Anthesis represents fully opening flowers. Three DAA-U, 6 DAA-U, and 9 DAA-U represent flower samples collected at 3, 6 and 9 d after anthesis, respectively, which were emasculated 2 days before opening. Three DAA-P, 6 DAA-P, and 9 DAA-P represent tomato flowers collected 3, 6, and 9 d after anthesis with hand pollination. ANOVA statistical analyses were performed using SPSS 15.0. Significant differences (*p* < 0.05) between treatments, as determined by Tukey’s tests, are indicated with different letters. Data are expressed as the mean ± standard errors for three replicates.

Expression analyses using real time-quantitative PCR (qRT-PCR) demonstrated that *SlARF5* was expressed in all tested organs or tissues, with relatively high levels in leaves and stems and significantly low levels in young ovaries before anthesis (Fig. 1A). At different developmental stages of the ovaries and in early-developing fruits, *SlARF5* expression decreased with flower development and reached its lowest levels in 6.0–7.5 mm ovaries just before anthesis (Fig. 1B). After pollination and fertilization, *SlARF5* expression was maintained at low levels, increasing only slightly at 6 days after anthesis (with hand-pollination) and then decreasing again at 9 days. However, when the flowers were emasculated at 2 days before anthesis, transcription levels increased markedly at anthesis, peaked at 3 days after anthesis (without hand pollination), and then decreased significantly from 6 to 9 days after anthesis. In general, *SlARF5* expression was downregulated in ovaries that were pollinated compared to that in ovaries from plants subjected to emasculation. Similar to *SlARF5*, pollination also suppressed the expression of *ARF8* in *Arabidopsis* and tomato^16^ and *SlARF7* in tomato^39^.

### Suppression of *SlARF5* induces parthenocarpy

**Figure 2:**
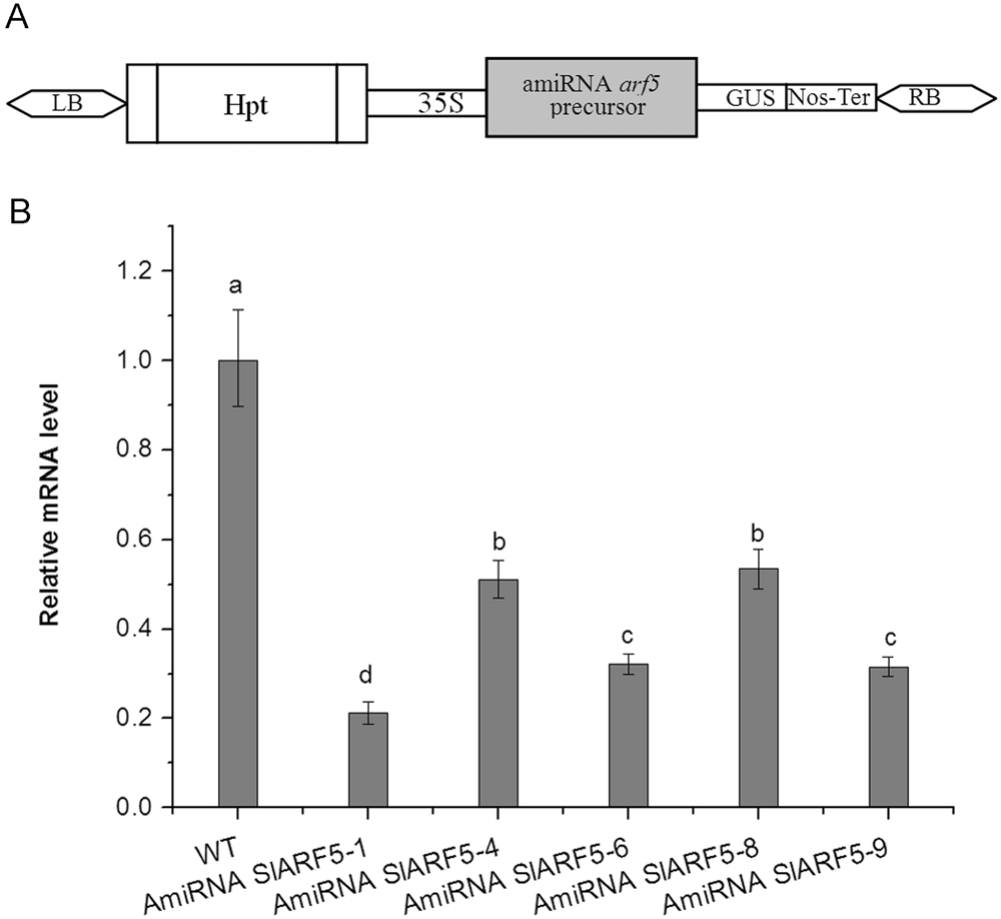
Construction of the *amiSlARF5* vector and identification of amiRNA transgenic tomato plants. (**A**) Schematic diagram of the *amiSlARF5* vector. The construct is under the control of the CaMV 35 S promoter and terminator and contains a selective marker for hygromycin antibiotic resistance. (**B**) Relative *SlARF5* mRNA levels in *amiSlARF5* and wild-type plants analyzed by qRT-PCR. ANOVA statistical analyses were performed using SPSS 15.0. Significant differences (P < 0.05) between treatments, as analyzed by Tukey’s test, are indicated as different letters. Data are expressed as the mean ± standard errors for three replicates.

To explore the function of SlARF5 during fruit set and development, *amiSlARF5* transgenic plants were generated using *A. thaliana* miRNA160a as a backbone to express an artificial miRNA (amiRNA) using a 125 bp *SlARF5*-specific fragment, which was cloned into the pCAMBIA1301-35S vector. The amiRNA binary vector was transferred into tomato using *Agrobacterium tumefaciens* (Fig. 2A). A total of 11 independent transgenic lines were generated and identified based on PCR and GUS screening (Supplementary Fig. S1). Among them, five lines exhibited significantly lower *SlARF5* expression levels compared to those in wild-type plants. In two *amiSlARF5* lines, namely, *SlARF5-*6 and *SlARF5-9*, the *SlARF5* transcription levels were reduced by 67.8% and 68.5%, respectively (Fig. 2B). In addition, although *SlARF6*, *SlARF7*, *SlARF8*, and *SlARF19* were closely related with *SlARF5* in molecular phylogenetic tree (Supplementary Fig. S2), their expression levels were not evidently affected in these two lines (Supplementary Fig. S3). Thus, these two *amiSlARF5* lines were used for further analysis.

**Figure 3:**
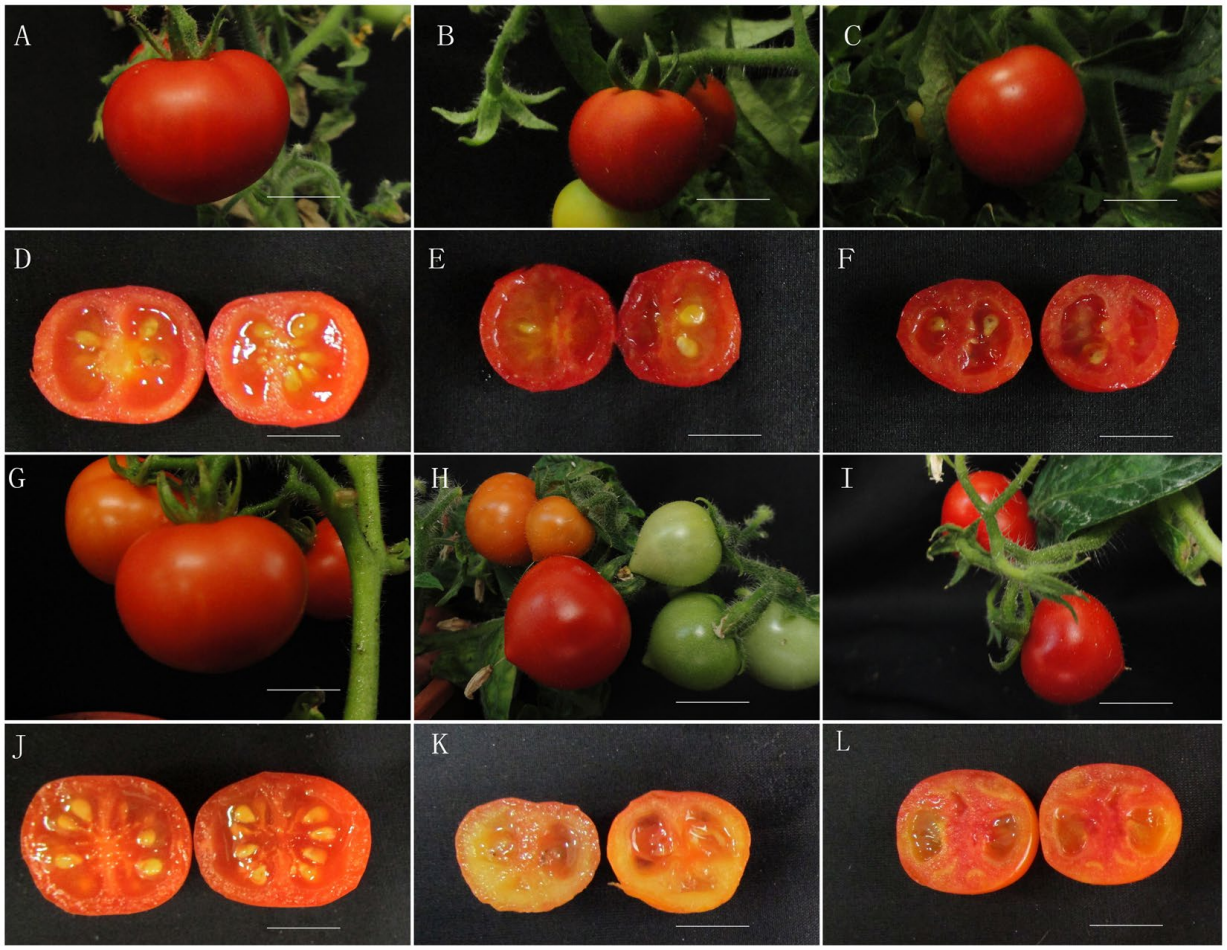
Phenotypic characteristics of *amiSlARF5* transgenic lines and control plants. (**A–C**) Self-pollination fruits of pCAMBIA1301 empty control (**A**), *amiSlARF5*-6 (**B**), and *amiSlARF5*-9 (**C**) transgenic plants. (**D**–**F**) Vertical section of the corresponding fruits from A, B, C, respectively; Bar = 1 cm. (**G**–**L**) Parthenocarpic fruits of *amiSlARF5* transgenic plants after emasculation compared to the pollinated control. (**G** and **J**) wild-type fruit and section; (**H** and **K**) *AmiSlARF5*-6 fruit and section; (**I** and **L**) *AmiSlARF5*-9 fruit and section. Bar = 1 cm.

No evident differences in vegetative growth or floral morphology were observed between *amiSlARF5* and wild-type plants (Supplementary Fig. S4). Furthermore, the fruit set rates in the *amiSlARF5*-6 and *amiSlARF5*-9 lines, 65.0% and 68.7%, respectively, were comparable to those of wild-type plants (74.2%) (Table 1). However, the self-pollinated *amiSlARF5* fruits were significantly smaller than wild-type fruits (Fig. 3A–F, Table 1). Both the polar and equatorial diameter of the transgenic fruits were much smaller compared to those obtained from wild-type and empty vector plants. Similarly, the weights of pollinated amiSlARF5 fruits (1.83 ± 0.27 g for *SlARF5*-6 and 1.84 ± 0.28 g for *SlARF5*-9) were much lower than those of the wild-type and empty vector transgenic fruits, at 3.40 ± 0.97 g and 3.32 ± 0.59 g, respectively.

**Table 1:** Characterization of fruit and seed development in wild-type tomato and *amiARF5* transgenic lines.

Line	Fruit weight (g)	Polar diameter (cm)	Equatorial diameter (cm)	Seeds/fruit	1000-seed weight (g)	Germination rate (%)	Fruit set rate (%)
Wild type	3.4 ± 0.97 a	1.9 + 0.12 a	2.02 ± 0.27 a	20.5 ± 4.9a	2.40 ± 0.24 a	70a	74.2 (n = 90)
1301	3.32 ± 0.59 a	1.84 ± 0.13 a	1.96 ± 0.13 a	18.5 ± 2.1a	2.50 ± 0.23 a	68.2a	70.1 (n = 75)
*ARF5*-6	1.83 ± 0.27 b	1.42 ± 0.33 b	1.49 ± 0.18 b	8.0 ± 2.8 b	2.36 ± 0.19 a	65.5a	65.0 (n = 85)
*ARF5*-9	1.84 ± 0.28 b	1.43 ± 0.13 b	1.44 ± 0.15 b	8.5 ± 0.7 b	2.37 ± 0.03 a	66.7a	68.7 (n = 70)
*ARF5*-6 (emasculation)	1.54 ± 0.44 c	1.34 ± 0.26 bc	1.36 + 0.39 c	0 c	—	—	20.5 (n = 75)
*ARF5*-9 (emasculation)	1.30 ± 0.42 c	1.22 ± 0.25 c	1.32 ± 0.27 c	0 c	—	—	22.4 (n = 90)
Wild type (emasculation)	—	—	—	—	—	—	0 (n = 75)

Note: The fruits were from mature fruits of pollinated and emasculated (indicated), approximately corresponding to 45 d after anthesis with pollination and unpollination, respectively. For each line, 15–30 fruits were sampled for statistical analysis. The total number of flowers for evaluation of fruit set rate is indicated (n).

Emasculation of untransformed ‘Micro-Tom’ plants 2 d before anthesis negatively affected fruit growth. Emasculated ovaries stopped growing 6 d after anthesis, became abortive and ultimately the flowers abscised within 6–9 days (Table 1). However, when the transgenic flowers were emasculated, the parthenocarpic fruit set and growth occurred in *SlARF5-*6 and *SlARF5-9* lines with fruit set rates of approximately 20.5% and 22.4% in these two transgenic lines, respectively. Although the fruit set rate in emasculated transgenic plants was lower than that in self-pollinated plants, suppression of *SlARF5* evidently initiated fruit development and enhanced parthenocarpic capability. However, the average size and weight of the parthenocarpic fruits were significantly lower than those of self-pollinated fruits. Consistently, Mapelli *et al*.^22^ also revealed that parthenocarpic fruits were generally smaller than seeded fruits. Furthermore, the smaller fruits in the *SlARF5 ami*RNA transgenic lines formed fewer locular tissues with reduced pulp and enlarged central columella compared to those observed in the wild-type fruits, resulting in parts of the locular cavities being empty (Fig. 3J–L). Interestingly, in parthenocarpic *SlARF7* RNAi fruits^39^ and in the fruits treated with GA_3_^40^, the locular tissue was barely developed, resulting in almost empty locular cavities.

Unexpectedly, self-pollinated transgenic fruits formed well-developed seeds of normal size and with germination potential; however, each fruit contained much lower numbers of fully developed seeds, 8.0 and 8.5 in *SlARF5*-6 and *SlARF5*-9 per fruit, respectively, compared to 20.5 and 18.5 in wild-type and empty-vector fruits, respectively (Table 1). The parthenocarpic fruits in the *amiSlARF5* lines had no seeds (Fig. 3K,L).

### Suppression of *SlARF5* expression affects cell division and expansion during early fruit development

In tomato, the cell division period lasts for 10–14 d. In the next 6–7 weeks, cell division activity is weakened and fruit growth set is predominantly dependent on cell expansion^22,24,41^. Our study demonstrated that transgenic plants of the *SlARF5*-6 and *SlARF5*-9 lines, in which *SlARF5* transcript levels were downregulated, produced smaller fruits than those of wild-type plants after pollination.

**Figure 4:**
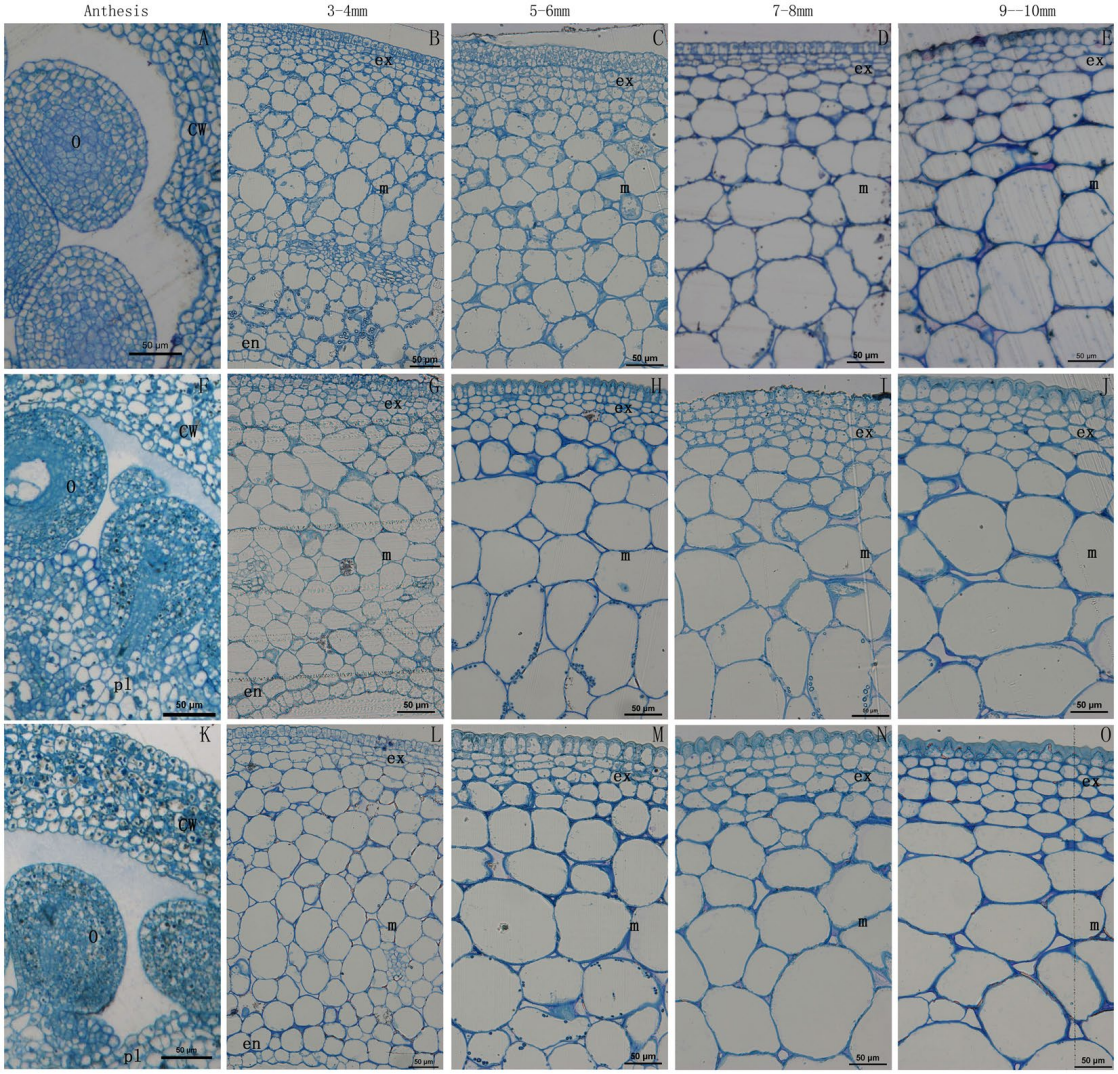
Microscopic analysis of the pericarp in the fruits of wild-type and transgenic lines during early tomato fruit development. (**A**–**E**) Wild-type fruit; (**F**–**J**) Transgenic line *ami*RNA *SlARF5*-6 fruit; (**K–O**) Transgenic line *ami*RNA *SlARF5-9* fruit. (**A**,**F**,**K**) Unpollinated ovary at the anthesis stage; (**B**,**G**,**L**) Pericarp of a 3–4 mm diameter fruit; (**C**,**H**,**M**) Pericarp of a 5–6 mm diameter fruit; (**D**,**I**,**N**) Pericarp of a 7–8 mm diameter fruit; (**E**,**J**,**O**) Pericarp of a 9–10 mm diameter fruit. Bars = 50 μm.

To understand the cause of different fruit phenotypes, histological cross-sections of wild-type and *SlARF5* transgenic plants were analyzed using the ovaries and developing fruits collected at diverse developmental stages, ranging from unpollinated ovaries to fruits 10 mm in diameter (Fig. 4A–O). In general, the pericarps of both the tomato fruits differentiated into the three classic layers: exocarp, mesocarp, and endocarp^24^. At anthesis, cell size in the pericarps of both wild-type and transgenic ovaries showed no obvious differences (Fig. 4A,F,K). In 3–4 mm fruits, the exocarp cells were at the stage of cell division, whereas the mesocarp and the endocarp cells began to expand. At this stage, the cell size in the mesocarps and endocarps of wild-type and transgenic lines were not significantly different. However, in 5–6 mm fruits, the exocarp and mesocarp cells in transgenic lines were significantly larger than those in wild-type plants. The mean cell area of the exocarp in RNAi fruits was nearly 2-fold greater than that of the wild-type fruits, while mesocarp cells were three times larger than those of wild-type cells (Fig. 4C,H,M, Table 2). The differences in exocarp features of RNAi and wild-type fruits were more clearly observed in the 7–8 mm and 9–10 mm fruits.

**Table 2:** Cell mean area of the pericarp of wild-type and transgenic fruits.

Line	Fruit size in	Exocarp	Mesocarp
	diameter (mm)	Mean area (μm^2^)	Mean area (μm^2^)
Wild type	5–6	163.0 ± 9.2 d (n = 1015)	1601.0 ± 148.8 e (n = 1145)
*ARF5*–6	5–6	322.9 ± 14.6 c (n = 1177)	4968.7 ± 287.7 cd (n = 735)
*ARF5*-9	5–6	376.1 ± 11.4 bc (n = 1425)	4415.0 ± 14.8 d (n = 892)
Wild type	7–8	385.4 ± 36.2 bc (n = 1200)	4410 ± 237.2 d (n = 1100)
*ARF5*-6	7–8	403 ± 34.2 bc (n = 1342)	5470.5 ± 186.9 bc (n = 720)
*ARF5*-9	7–8	536.5 ± 37.9 a (n = 630)	5975 ± 635.8 ab (n = 510)
Wild type	9–10	468.8 ± 61.4 ab (n = 645)	5213.6 ± 235.0 bcd (n = 460)
*ARF5*-6	9–10	554.6 ± 54 a (n = 922)	6792 ± 315.9 a (n = 475)
*ARF5*-9	9–10	552.9 ± 32 a (n = 810)	6533.8 ± 546.8 a (n = 532)

Wild-type and transgenic fruits of 5–6 mm, 7–8 mm, and 9–10 mm diameter, were used for the measurement of cell area (in μm^2^). Approximately 100 cells for exocarp or mesocarp per fruit were analyzed at early stage, whereas more than 30 cells at late stage. The total number of cells each line for analysis is indicated (n). The Data represent the means ± standard error of five fruits from each transgenic at each development stage with three replicates. The different letters indicate the significant differences between wild-type and transgenic fruits (*p* < 0.05, Student’s t test).

**Figure 5:**
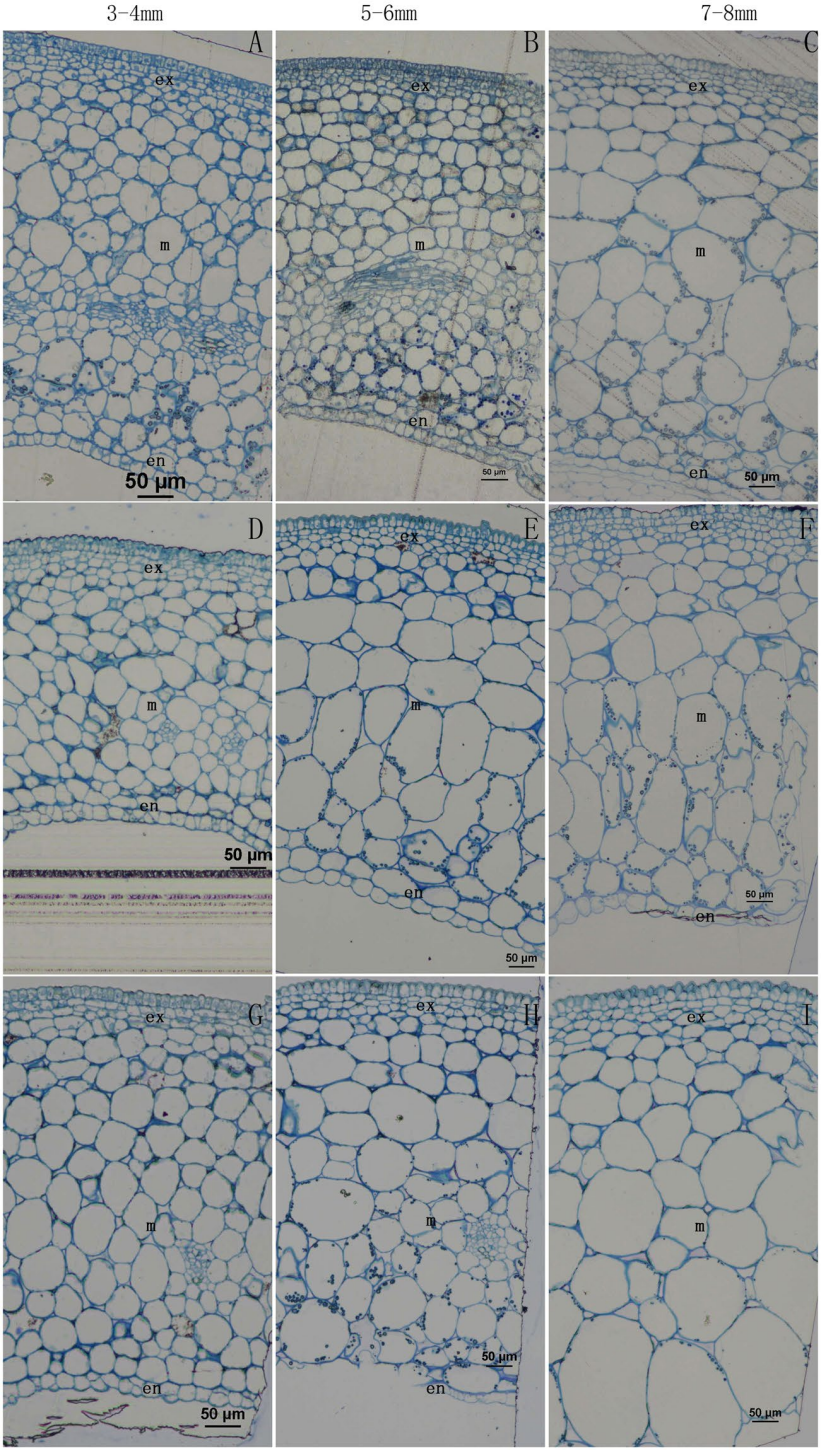
Micrograph of the number of cell layers in wild-type and transgenic line fruits during early fruit development. (**A**,**D**,**G**) 3–4 mm diameter fruits of wild-type (**A**), *ami*RNA *SlARF5-6* (**D**), and *amiSlARF5*-9 plants (**G**). (**B**,**E**,**H**) 5–6 mm diameter fruits of wild-type (**B**), *amiSlARF5*-6 (**E**), and *amiSlARF5*-9 plants (**H**). (**C**,**F**,**I**) 7–8 mm diameter fruits of wild-type (**C)**, a*miSlARF5*-6 (**F**) and *amiSlARF5*-9 plants (**I**). Bars = 50 μm.

On the other hand, there are significant differences in the number of cell layers between transgenic *amiSlARF5* lines and wild-type plants. In general, the exocarp cells of tomato fruit comprise 4–5 cell layers outside the fruit, and the endocarp cells comprise 1–2 cell layers inside the fruit; therefore, the mesocarp cell number was the major contributor towards the differences in fruit equatorial diameter among the plants with different genotypes. In 3–4 mm fruits, pollinated transgenic fruits formed fewer cell layers (approximately 16 cell layers) than those in the wild-type plants (at least 23 cell layers), a reduction of 30% compared to wild-type plants. Similarly, in the 5–6 mm and 7–8 mm fruits, the pollinated transgenic fruit formed fewer cell layers than those in the wild-type fruit, with levels being 32% and 25–31% lower, respectively (Fig. 5A–I, Table 3). This indicates that inhibition of *SlARF5* leads to a significant reduction in the number of cell layers. The small size of mature transgenic fruits may be primarily attributed to the reduced number of cell layers in transgenic fruits, probably as a result of a reduction in the period of cell division that promotes cell layer formation.

**Table 3:** Number of cell layers in wild-type and *SlARF5* transgenic fruits.

Line	Fruit diameter (mm)		
	3–4	5–6	7–8
Wild type	23 ± 2.83 a	25.17 ± 3.31 a	21.5 ± 1.87 a
*amiSlARF5*-6	16 ± 1.15 b	17.83 ± 0.58 b	16.17 ± 1.17 b
*amiSlARF5*-9	16.33 ± 1.75 b	17 ± 1.41 b	15 ± 2.09 b

Wild-type and transgenic fruits of 3–4 mm, 5–6 mm, and 7–8 mm diameter were used for estimating the number of cell layers. Data represent the means ± standard error of five fruits per transgenic line at each development stage with three replicates. Different letters indicate the significant differences between wild-type and transgenic fruits (*P *< 0.05, Student’s t test).

### Transcriptomic analysis of the *amiSlARF5* and WT fruits

To evaluate expression changes during fruit development in *amiSlARF5*-9 transgenic and wild-type plants, massively parallel RNA sequencing (RNA-Seq) analyses were carried out using the RNA samples from 3–4 mm young fruits with two replicates. At this stage, *SlARF5* was highly expressed in the wild type and no phenotypic differences were observed between *amiSlARF5* and wild-type fruits. We generated four cDNA libraries from each genotype for sequencing using the Illumina Hiseq. 2000/2500 system at LC Sciences (Hangzhou, China). Each cDNA library yielded more than 45 million sequence reads (Supplementary Table S1), representing >4 Gb sequence data per sample. RNA data from the two biological replicates exhibited good correlations and were used for further analysis (Supplementary Fig. S5). A summary of RNA sequencing, mapping, assembly and annotation is provided in Supplementary Table S1.

**Figure 6:**
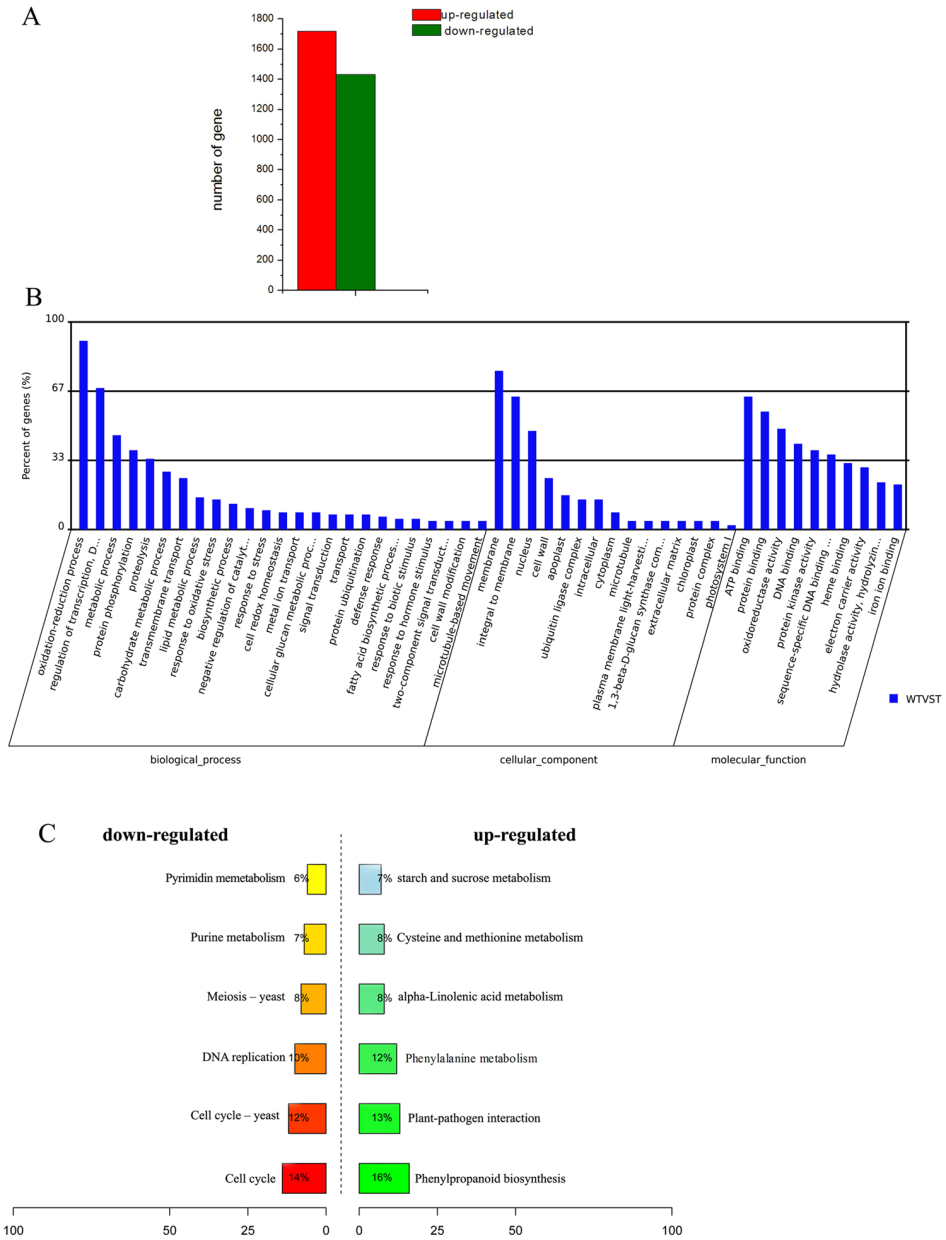
Analysis of genes regulated by *SlARF5* during fruit development. (**A**) The numbers of differentially expressed genes in tomato fruit after *SlARF5* expression was inhibited. (**B**) Analysis of biological functions of GO-enriched genes. (**C**) The primarily enriched KEGG pathway for up- and downregulated differentially expressed genes.

Compared to wild-type fruits, a total of 3152 genes were differentially expressed in *amiARF5* transgenic plants, with 1719 genes upregulated and 1433 genes downregulated (*p* < 0.05, |log_2_(fold changes)| ≥2) (Fig. 6). As expected, *SlARF5* was downregulated by approximately 16-fold in transgenic plants. Seventeen genes from the RNA-seq-derived results were validated by qRT-PCR. The results demonstrated that almost all tested genes were in agreement with those from the RNA-seq data (Supplementary Fig. S6), confirming the reliability of RNA-seq analysis.

To further evaluate the potential functions of differentially expressed genes (DEGs) in the transgenic fruits, functional enrichment analysis was performed. GO analysis indicated that these DEGs were primarily enriched in ATP, protein, or DNA binding (383), protein kinase activity (147), oxidoreductase activity (131), and catalytic activity (60), which is consistent with kinase genes associated with clusters that are highly expressed in young fruit^42^. Regarding the biological processes, a high proportion of DEGs was associated with oxidation-reduction processes, regulation of transcription, protein phosphorylation and metabolic metabolism. Among them, transcription regulation, signal transduction, and metabolic processes accounted for more than half of all annotated DEGs, implying that signal transduction pathways and metabolic activities are strongly affected by inhibition of the *SlARF5* gene. In addition, in terms of the cellular components, most DEGs were primarily enriched in the membrane, nucleus, or the cell wall (Fig. 6), which is consistent with reports demonstrating that the cell wall plays essential roles in fruit set and fruit size^43^. Similarly, previous transcriptome analysis indicated that the cell-cycle and cell-wall genes were enriched in ovule/seed-associated expression clusters^42^.

KEGG analysis further indicated that most of the downregulated genes are involved in the cell cycle (14%), the cell cycle of yeast (12%), and DNA replication (10%) in *amiSlARF5* transgenic fruits, suggesting that SlARF5 is positively involved in cell division. The upregulated genes were associated with phenylalanine metabolism (12%), phenylpropanoid biosynthesis (16%), plant-pathogen interactions (13%), and starch and sucrose metabolism (7%) (Fig. 6). Among these DEGs, the expression of several genes related to fruit development was significantly altered in transgenic plants, including those involved in cell division, cell expansion, and hormone-signaling pathways (Tables 4 and 5 and Fig. 6).

**Table 4:** Differentially expressed genes involved in cell cycle and cell wall development.

Gene ID	Gene Description	T_fpkm	WT_fpkm	log_2_ (Fold changes)	*p*-value
**Cell cycle**					
Solyc11g005090.1	*Cyclin A1 (CycA1)*	10.36	33.01	−1.67	0.02
Solyc06g065680.2	*Cyclin A2 (CycA2)*	12.02	23.7	−0.98	0.02
Solyc10g078330.1	*B-type cyclin cycb1d1 (cycB1.1)*	11.08	26.09	−1.23	0.01
Solyc01g009040.2	*CyclinB1-4 (CycB1-4)*	12.93	29.42	−1.19	0.01
Solyc04g082430.2	*CyclinB2-4 (CycB2-4)*	12.94	25.64	−0.99	0.02
Solyc01g089850.2	*CyclinU4-1 (CycU4-1)*	9.97	57.04	−2.52	5E-05
Solyc01g107730.2	*D-type cyclin-2 (CycD3;2)*	34.34	73.59	−1.1	0.01
Solyc04g078470.2	*D-type cyclin-3 (CycD3;3)*	6.08	30.49	−2.33	5E-05
Solyc10g074720.1	*B1-type cyclin dependent kinase (CDKB1)*	18.59	44.79	−1.27	0.01
Solyc04g082840.2	*B2-type cyclin dependent kinase (CDKB2)*	44.87	90.11	−1.01	0.02
Solyc06g076660.2	*Proliferating cell nuclear antigen gene (PCNA)*	32.56	94.27	−1.53	0
Solyc03g113760.2	*E2F transcription factor-like protein (E2FE)*	3.75	9.04	−1.27	0.02
Solyc06g008030.2	*phytochrome interacting factor 3-like 5 (PIF1)*	13.01	3.98	1.71	0.05
**Cell wall**					
Solyc01g109790.2	*ADP-glucose pyrophosphorylase (AGPS1)*	41.76	129.32	−1.63	0
Solyc08g005800.2	*Pectinacetylesterase like protein (PAE)*	395.18	115.08	1.78	0
Solyc07g017600.2	*Pectin methylesterase 44 (PME44)*	121.42	61.53	0.98	0.03
Solyc06g049050.2	*Expansin 2 (EXP2)*	33.18	129.38	−1.96	0
Solyc05g007830.2	*Expansin 12 (EXP12)*	76.99	236.99	−1.62	0
Solyc01g081060.2	*Xyloglucan endotransglucosylase-hydrolase 14 (XTH14)*	10.32	3.86	1.42	0.01
Solyc12g011030.1	*Xyloglucan endotransglucosylase-hydrolase 9 (XTH9)*	20.34	44.04	−1.11	0.03
Solyc03g031800.2	*Xyloglucan endotransglucosylase/hydrolase 1(XTH1)*	44.08	103.44	−1.23	0.01
Solyc11g008820.1	*Endoglucanase*	5.03	19.33	−1.94	0
Solyc08g083210.2	*endo-1,4-beta-glucanase (CEL5)*	0.44	7.64	−4.12	0
Solyc07g052510.2	*Cell wall biosynthesis, Peroxidase (TPX1)*	23.88	58.48	−1.29	0
Solyc12g099200.1	*Homolog to invertase/pectin methylesterase inhibitor*	224.72	109.48	1.04	0.01

**Table 5:** Differentially expressed genes involved in auxin and gibberellin signaling.

Gene ID	Gene Description	T_fpkm	WT_fpkm	log_2_ (Fold changes)	*p*-value
**Auxin-related genes**					
Solyc01g111310.2	*Auxin Efflux Facilitator (LAX2)*	11.01	25.92	−1.24	0
Solyc02g037550.2	*Auxin efflux carrier family protein*	12.14	36.84	−1.6	0
Solyc12g014500.1	*IAA carboxylmethyltransferase 1 (IAMT1)*	0.4	10.02	−4.65	0.01
Solyc09g090910.1	*Auxin-induced protein 13 (IAA13)*	8.74	25.32	−1.53	0
Solyc09g083290.2	*Indole-3-acetic acid inducible 14 (IAA14)*	4.85	11.4	−1.23	0.04
Solyc09g083280.2	*AUX/IAA transcriptional regulator family protein* *(IAA1)*	10.35	24.45	−1.24	0.01
Solyc09g064530.2	*Auxin-induced protein 12 (IAA12)*	6.26	29.27	−2.22	0
Solyc07g008020.2	*Indole-3-acetic acid inducible 35 (IAA35)*	5.61	15.42	−1.46	0.02
Solyc06g008590.2	*Indoleacetic acid-induced protein 17 (IAA17)*	6.84	43.15	−2.66	0
Solyc06g008580.2	*AUX/IAA transcriptional regulator family protein (IAA22)*	1.03	11.55	−3.49	0.01
Solyc04g081240.2	*Auxin Response Factor 5 (SlARF5)*	0.95	14.26	−3.9	5.00E-05
Solyc06g084070.2	*AUX/IAA gene family protein (IAA2)*	0.81	14.07	−4.12	0
Solyc09g083290.2	*AUX/IAA gene family protein (IAA24)*	4.85	11.4	−1.23	0.04
Solyc03g121060.2	*AUX/IAA gene family protein (IAA18)*	40.95	20.48	1	0.03
Solyc08g082630.2	*Auxin Response Factor 9 A (SlARF9A)*	5.54	33.12	−2.58	5.00E-05
Solyc09g083290.2	*AUX/IAA gene family protein (SlIAA14)*	4.85	11.4	−1.23	0.04
Solyc01g107390.2	*Auxin- and ethylene-responsive GH3-like protein* *(GH3.1)*	3.48	1.42	1.3	0.05
Solyc06g062920.2	*Auxin-regulated dual specificity cytosolic kinase*	23.67	2.69	3.14	5.00E-05
Solyc06g075690.2	*Auxin-regulated protein AF416289*	49.05	2.97	4.05	5.00E-05
**Gibberellin-related genes**					
Solyc03g006880.2	*GA 20-oxidase-oxidase-1 (SlGA20ox1)*	0.14	36.35	−8.02	0.03
Solyc04g078390.1	*F-box family protein (SLY1)*	131.09	59.27	1.15	0
Solyc07g054310.1	*Cell wall protein (GA-induced)*	789.25	204.58	1.95	0
Solyc09g074270.2	*Alpha/beta-Hydrolases superfamily protein (GID1B)*	28.92	10.95	1.4	0
Solyc02g083880.2	*Gibberellin-regulated family protein* (s*imilar to GASA2/GAST1 protein in Arabidopsis*)	474.19	55.53	3.09	0.00005
Solyc01g10737 Solyc01g107370.2	*Gibberellin-regulated family protein (similar to cyclin-D3-1gene in Arabidopsis)*	0.49	8.56	−4.12	0.05
Solyc12g042500.1.1	*Gibberellin-regulated family protein (similar to Cyclin-T1-2 in Arabidopsis)*	36.76	95.5	−1.38	0.04
Solyc03g116060.2	*Gibberellin-regulated family protein (similar* to *Gibberellin-regulated family protein in Arabidopsis)*	2.75	20.2	−2.88	0

### Cell division- and cell expansion-related genes

As shown in Table 4, 12 cell cycle-related genes, including *cycA1, cycA2, cyclin B1, cycd3C2/C3*, and *cyclin-dependent protein kinases*, and eight cell wall development- related genes, including *EXP2, EXP8, xyloglucan, pectinacetylesterase*, and *endoglucanase*, were downregulated in the transgenic lines compared to the levels observed in wild-type plants, suggesting that both fruit cell division and expansion is negatively affected in *ami*RNA *SlARF5* transgenic plants.

**Figure 7:**
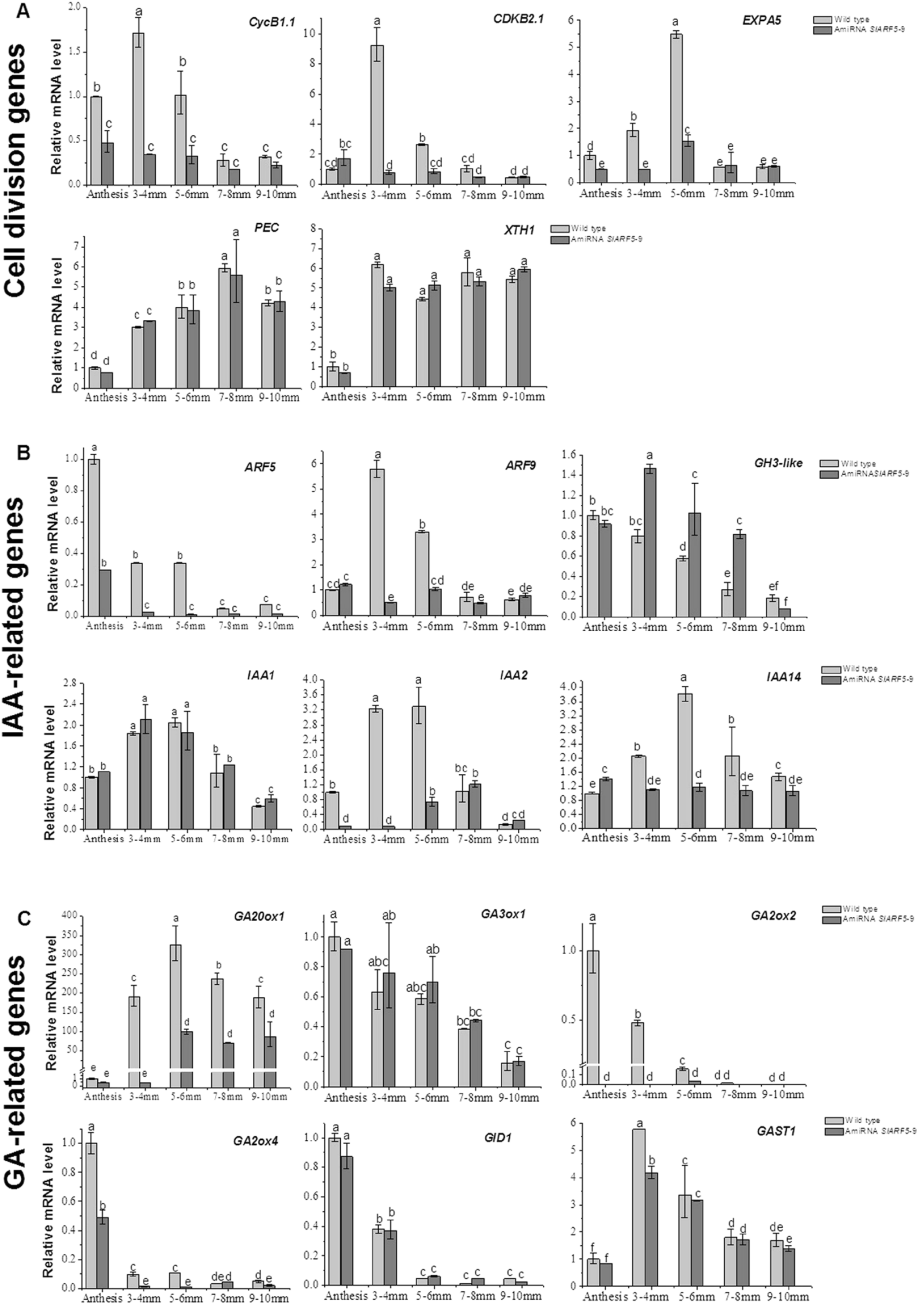
Expression of differentially expressed genes in developing fruits of wild-type and transgenic plants by qPCR. (**A**) Cell division and cell expansion genes*-CycB1.1*, *CDKB2.1, EXPA5, PEC* and *XTH1*. (**B**) Auxin-related genes-*ARF5*, *ARF9*, *GH3-like, IAA1*, *IAA2* and *IAA14*. (**C**) Gibberellin-related genes-*GA20ox1*, *GA3ox1*, *GA2ox2*, *GA2ox4*, *GID1* and *GAST1*. Ovaries and young fruits of WT and amiRNA plants were sampled at anthesis, and at 3–4 mm, 5–6 mm, 7–8 mm, and 9–10 mm diameter stages. Significant differences (P < 0.05) between treatments, as analyzed by Tukey’s test, are indicated as different letters. Data are expressed as the means ± standard errors for three replicates.

To explain the histological data obtained from microscopic analysis in relation to RNA-Seq results, the ovaries and fruits were collected from wild-type and transgenic plants for RNA extraction to analyze expression patterns of cell expansion- and division-related genes. In the wild-type fruits, the transcript levels of cell cycle genes, including *SlCyclinB1.1* and *SlCDKB2.1*, were markedly increased following anthesis, and then decreased continuously during later fruit development. However, in transgenic lines, these cell cycle-related genes (*CycB1.1* and *CDKB2.1*) were significantly downregulated in the early stages of fruit development compared to the levels observed in wild-type fruits (Fig. 7A). In the later stages of fruit development, i.e., 7–8 mm fruits and above, there were no significant differences in the expression patterns of the cell cycle genes between the transgenic and wild-type fruits.

Similarly, in wild-type fruits, the expression of cell expansion-related genes (i.e., *SlEXPA5*, *SlPEC*, and *SlXTH1*) increased significantly following anthesis during early fruit development. In the 3–4 mm and 5–6 mm fruits, the expression of *SlEXPA5* in transgenic plants was significantly lower than that in wild-type fruits, and in the 7–8 mm fruits and later stages, the transcript levels of *SlEXPA5* in transgenic fruits were similar to those in wild-type fruits. However, the mRNA levels of *SlPEC* and *SlXTH1* in both transgenic and wild-type fruits were maintained at similar levels during early fruit development (Fig. 7A). These results suggest that the microscopic characteristics of the transgenic lines are primarily due to reduced levels of cell division-related genes.

### Auxin-signaling pathway genes

Previous studies have confirmed that auxin stimulates cell division^40,41^. As shown in Table 5, 15 genes involved in auxin homeostasis, transport and signaling, including *LAX2*, *ARF5*, *SlARF9*, *IAA1*, *IAA2*, and *IAA14*, were downregulated in the transgenic lines compared to the levels observed in wild-type plants, suggesting that IAA-related genes are negatively affected in *ami*RNA *SlARF5* transgenic plants. In wild-type plants, the mRNA levels of some auxin response genes (e.g., *SlARF9, SlIAA1, SlIAA2, and SlIAA14*) increased in 3–4 mm fruits and then decreased to significantly low levels during later stages. However, in transgenic plants, the mRNA levels of *SlARF9*, *SlIAA2*, and *SlIAA14* were downregulated compared to those in wild-type plants, whereas *SlIAA1* was maintained at similar mRNA levels as those in wild-type plants. As expected, the expression of *SlARF5* in transgenic plants was significantly lower than that in wild-type plants in the early stages, up to the 5–6 mm fruit stage. Similarly, all detected auxin-responsive genes, including *SlARF9*, *SlGH3*, *SlIAA1*, and *SlIAA2*, were significantly downregulated in the early development stage compared to the levels observed in wild-type plants, with the exception of a GH3-like gene, which was significantly upregulated in the 3–4 mm and 5–6 mm fruit stages (Fig. 7B); these results are consistent with the RNA-seq data, thereby suggesting that suppression of SlARF5 strongly affects several genes related to the auxin-signaling pathway. Unlike previous microarray data^43^, in which the expression levels of most auxin-related genes were increased in the pollinated fruit, our RNA-Seq results demonstrated that a majority of the genes involved in the auxin signaling pathway were downregulated, in alignment with the microarray data of GA-treated fruit^43^.

### Gibberellin-related genes

The GA-biosynthesis gene, *GA20ox1*, was significantly downregulated in the transgenic lines compared to levels observed in wild-type plants (Fig. 7C), whereas four GA-related genes, including *SLY1, GID1B, Cell wall protein* (*GA-induced*), and GA-regulated family protein, were upregulated in the transgenic lines compared to the levels in wild-type plants (Table 5), thereby suggesting that GA-biosynthesis genes are negatively affected in *ami*RNA *SlARF5* transgenic plants, whereas certain GA-signaling genes are upregulated. Similarly, qRT-PCR analysis demonstrated that in wild-type plants, the expression levels of *SlGA20ox1* and *SlGAST1* markedly increased following pollination, and progressively decreased thereafter. However, *SlGA2ox2, SlGA3ox1, SlGA2ox4*, and *SlGID1* continued to decline following anthesis during fruit development. In the *ami*RNA *SlARF5* lines, *SlGA3ox1*, *SlGID1*, and *SlGAST1* exhibited similar expression patterns as those in wild-type plants, with no significant differences between each successive stage of early fruit development, with the exception of *SlGAST1* levels at the 3–4 mm stage. However, the expression level of *SlGA20ox1* was significantly downregulated in all successive stages following anthesis, whereas *SlGA2ox2* and *SlGA20ox4* were significantly downregulated at the anthesis, 3–4 mm and 5–6 mm stages. During later stages, the differences in expression levels became non-significant in both transgenic lines and wild-type plants (Fig. 7C). Previous microarray data^43^ also demonstrates increased the expression of most GA-signaling genes in GA_3_-treated ovaries.

## Discussion

In plants, ARFs encode important transcription factors and regulate gene expression in response to auxin by binding specifically to the TGTCTC sequence in the auxin response elements found in the promoters of primary/early auxin response genes. Recently, all ARF family genes were identified in tomato^44^, an ideal model plant for studying fruit development and ripening. However, only SlARF6, SlARF7, SlARF8, and SlARF9 have been characterized to date and have been demonstrated to be essential for tomato fruits generated through the regulation of auxin signal transduction. In this study, transgenic plants with reduced *SlARF5* expression were developed using artificial miRNA silencing methods in order to examine the role of SlARF5 in fruit initiation and development by affecting the IAA and GA signaling pathways.

### Suppressing expression of *SlARF5* in tomato induces parthenocarpic fruit

Previous studies have demonstrated that SlARF8 is a negative regulator of fruit initiation in *Arabidopsis*^16^. SlARF7 and SlARF8 affect fruit initiation and lead to parthenocarpy^16,39^. SlARF7 functions as a negative regulator of fruit set until pollination and fertilization^18^. In addition, SlARF9, which is enriched with serine, was reported to negatively regulate cell division during early fruit development^20^. Interestingly, these ARFs are typical tomato auxin response factors with a glutamine-rich middle region belonging to the class II subfamily, and the others being *Sl*ARF5, *Sl*ARF6-1, *Sl*ARF8-1, *Sl*ARF19, and *Sl*ARF19-1^38^. ARFs enriched with glutamine are likely to function as transcriptional activators^38^. It is worth noting that although these genes belong to the same subfamily, their expression patterns during fruit development were quite different. *SlARF5* mRNA levels were evidently higher in emasculated fruits than those in self-fertilized fruits. These results suggest that SlARF5 plays a role in fruit initiation when pollination occurs.

In *Arabidopsis*, ARF5 affects lateral organ development^45^, primary root initiation^46^, flower primordium initiation^47^, and cotyledon development in embryos^48^. Our data demonstrated that *ami*RNA *SlARF5* transgenic fruits were significantly smaller than wild-type fruits, and their average weight and equatorial and longitudinal diameter were significantly decreased compared to that of wild-type fruits (Table 1). Interestingly, the number of seeds in the self-fertilized transgenic lines was significantly lower than that in wild-type plants. However, when the transgenic flowers were emasculated, the *amiSlARF5* plants developed parthenocarpic fruits, and the parthenocarpic fruit set rate was approximately 20.5% and 22.4% in two independent transgenic lines. The average size and weight of the parthenocarpic fruits were significantly lower than those in self-pollinated fruits. In previous studies, downregulation of *SlDELLA*^49^, *TM29*^50^, *CHS*^51^ and *AUCSIA* genes^52^, mutations of *pat*^53^ and *hydra*^54^ and transformation of aberrant ARF8^19^ led to the formation of smaller parthenocarpic fruits, implying that these genes are positive regulators of fruit development in tomato. However, the parthenocarpic tomato fruits developed in *rolB*^55^, *SlARF7* RNAi^18^, *SlIAA9* knockout^56^ and *SlTIR1* over-expression transgenic plants^57^ were similar in size to those of wild-type plants. Interestingly, overexpression and inhibition of *SlARF9* mRNA levels led to smaller fruits and larger fruits, respectively^20^, suggesting different functions of ARFs in parthenocarpic fruit development.

### SlARF5 modulates the expression of cell division- and expansion-related genes and the crosstalk between auxin- and GA-signaling

The signal transduction mechanism by which auxin regulates cell division and cell expansion during fruit set and growth is largely unknown. Previous studies have demonstrated that suppression of *SlARF7* reduces cell division ability and enhances cell expansion ability^18^, whereas ARF6 may affect epidermal cell differentiation of petals in *Arabidopsis*^14^. In the present study, the inhibition of *SlARF5* reduced fruit cell division and expansion. RNA-Seq data demonstrated that the expression levels of most cell cycle genes were downregulated in the 3–4 mm diameter fruit stage. GO annotation and KEGG analyses indicated that the cellular components of cell walls and pathways of the cell cycle-related to fruit development were observed to be enriched. The cell cycle genes, *cycB1.1* and *CDKB2.1*, were significantly downregulated in *amiSlARF5* plants during early fruit development, compared to the levels in wild-type fruits, indicating that cell division activity in *amiSlARF5* fruits was decreased. However, in transgenic tomato plants with inhibition of *SlARF7* or *SlIAA9*, the expression of cell cycle-related genes was lower and higher than that in wild-type plants, respectively^39,58^.

Cell expansion genes are induced by pollination and GA treatment^43^. Expression of the cell expansion gene *EXPA5*, which is induced by pollination rather than by GA application, is downregulated during early fruit development, whereas *XTH1* and *PEC* are upregulated by GA application^43^. In our study, RNA-Seq data demonstrated that certain cell expansion genes (e.g., *EXP2/EXP12, AGPS1, CEL5*, and *TPX1*) in the *amiSlARF5* line were downregulated compared to the levels in wild-type plants, suggesting that cell expansion abilities in the ami*SlARF5* line were weakened. In a previous study, the mRNA levels of two cell expansion-related genes, namely, *SlPEC* and *SlXTH1* in the *SlARF7* RNAi line, were similar at anthesis but became higher than those in the wild-type plants following anthesis during early fruit development^39^.

Plant hormones, such as auxin and gibberellins, play pivotal roles in tomato fruit development. The IAA and GA pathways regulate the cell cycle and expansion genes and determine the final fruit size^24,40,41^. In normal fruit, there is a rapid increase in the GA levels induced by successful pollination and fertilization, following which GA may induce an increase in auxin levels during the first 10 DPA^22,59^. However, in *amiSlARF5* plants, a majority of the IAA-related genes were significantly downregulated, as demonstrated by the RNA-Seq data and further verified by qRT-PCR analysis. IAA-related genes, *SlARF9*, *IAA2* and *IAA14*, which are induced by pollination and auxin treatment^43^, were downregulated during early fruit development in *amiSlARF5* lines. However, expression of *GH3*, which is induced by auxin application to unpollinated ovaries^43^, increased in transgenic plants, as demonstrated by the qRT-PCR and RNA-Seq results, thereby suggesting that at least part of the IAA pathway was enhanced.

GA20ox1, an enzyme involved in GA biosynthesis, catalyzed the conversion of GA_12_ and GA_53_ into GA_9_ and GA_20_, respectively, which are precursors of bioactive GAs^60^. The expression of *GA20ox1* was significantly decreased in *amiSlARF5* lines during early fruit development compared to that in wild-type fruits, which is consistent with the reduction in parthenocarpic fruit size of *SlARF7* RNAi plants^18^. Similarly, the genes coding GA biosynthetic enzymes, GA2ox2 and GA2ox4, which catalyze active GA_1_ and GA_4_ conversion into inactive GA_34_ and GA_8_, were significantly downregulated in *amiSlARF5* lines. However, GA3ox1, involved in the conversion of GA_9_ and GA_20_ into GA_1_ and GA_4_, exhibited similar expression levels in transgenic and wild-type plants. On the other hand, GA-signaling transduction genes, such as *GID1*, were positively induced by GA treatment^59^. In our RNA-Seq data, the expression levels of *GID1* and certain other known GA-signaling and response genes in *amiSlARF5* lines increased compared to those in wild-type plants. These results suggest that levels of certain GA-signaling-related genes increased. This may be attributed to the feedback regulation on GA-signaling, which is similar with a previous study in *PttGID1* over-expression lines^61^. The more pronounced responses to GA compared to those of wild-type plants were found in over-expression lines, while *PttGA20ox1* expression was reduced^61^. Although three GA-regulated family proteins were downregulated in the *amiSlARF5* lines, their functions were undefined. Two of these genes were probably cell cycle family genes in *Arabidopsis* (Table 5), which may function as the downstream genes of GA signaling pathway. Consistently, in our RNA-Seq results, mRNA levels of most cell cycle genes were also downregulated (Table 4).

The DEGs between *amiSlARF5* transgenic and wild-type plants, identified by RNA-seq, were compared with the data from microarray analyses of GA_3_-treated and pollinated fruits (RNA from emasculated flowers)^43^. Our data demonstrated that the expression levels of most cell cycle and expansion genes were downregulated in transgenic plants, unlike their increased expression in GA_3_-treated and pollinated plants^43^. Furthermore, the alterations in the expression of some GA and IAA pathway genes in our study were in contrast to those observed by microarray analysis in pollinated fruits^43^; however, the altered levels of certain GA-related genes, such as *SlGA3ox1* and cell wall protein precursor in GA-treated fruits^43^, were similar with our data. These results suggest that the inhibition of *SlARF5* enhanced part of the GA pathway, similar to the results involving GA-treated fruits^43^.

Previous studies have indicated that the expression of GA metabolism genes is regulated by Aux/IAA and ARF proteins, and changes in GA metabolism partially mediate auxin action during development^62^. ARF7 and ARF19 serve as crosstalk between auxin signaling and in ethylene responses in *Arabidopsis*^63^. The RNA-seq results of *SlARF3* RNAi lines indicated that the *SlARF3* gene regulates the auxin-dependent transcription of trichome formation by mediating auxin, ethylene, and GA signaling in tomato^5^. Recently, Breitel *et al*.^64^ demonstrated that SlARF2 links the signals of auxin, ethylene, and other hormones and affects the ripening capacity of fruit tissue. In pollination-dependent and parthenocarpic fruit set, ARF2 and IAA9 mediate the crosstalk between auxin and GA during this development process^65^. In obligatory parthenocarpy caused by *SlMIR159*-overexpressing tomato cv. Micro-Tom plants, SlGAMYBs potentially contribute to fruit initiation and regulate auxin and gibberellin responses^66^.

In the *ami*RNA *SlARF5* lines, when the *SlARF5* mRNA was silenced, fruit set was evidently different from that observed in previous studies, in which the fruit set may be due to elevation of hormones (especially auxin) by expression of auxin biosynthesis genes in ovaries and ovules^55,67,68^. However, in our transgenic lines, GA signaling was potentially regulated by GA biosynthesis pathways (downregulated gene expression) through negative feedback, as a result, some genes involved in the GA-signaling pathway were upregulated. SlARF5 may function as SlARF7, which was suggested by De Jong *et al*.^39^. When SlARF5 was inhibited, the special downstream genes, such as auxin attenuating genes might fail to form transcripts; therefore, the inhibition imposed on ovary development was relieved, thereby enabling the ovary to expand and form the fruit. Meanwhile, in our transgenic plants, most of the auxin-related genes were not regulated and only *GH3* was increased in the *amiSlARF5* transgenic plants. The hormone-related gene expression was consistent with the *ARF7* RNAi results^18^. As a result, once *SlARF5* was inhibited, increased GA signaling gene expression may facilitate fruit development. On the other hand, the smaller fruit size in *amiSlARF5* plants may be attributed to the weakened auxin signaling pathway.

In conclusion, SlARF5 is a essential component of auxin signaling during tomato fruit development. SlARF5 may be critical for the crosstalk between auxin and GA during fruit development. In normal-pollination ovaries of wild-type plants, the mRNA level of *SlARF5* was decreased, and the auxin- and GA-signaling pathways converged at fruit set and development. However, in emasculated flowers of wild-type plants, the *SlARF5* mRNA levels were increased. Therefore, the *SlARF5* mRNA levels potentially negatively regulate the GA-signaling pathway. A partially enhanced GA-signaling pathway may lead to the formation of parthenocarpic fruit.

## Materials and Methods

### Plant materials and growth conditions

Tomato (*S. lycopersicum* L.cv. Micro-Tom) plants were grown in a standardized greenhouse at the experimental farm at Zhejiang University, under 14/10 h light/dark periods with 200 μmol m^−2^s^−1^ photo flux and 27/22 °C (day/night) temperatures.

For the measurement of early fruit development parameters, the flowers were emasculated 2 days before opening, then ovaries were collected at 0, 3, 6, and 9 DAA-U and DAA-P, and early developing fruits were collected in the following stages: pollinated ovaries at anthesis and 3–4 mm, 5–6 mm, 7–8 mm, and 9–10 mm diameter fruit stages, corresponding to approximately 6, 8, 10, and 12 DAA-P stages of the wild-type, respectively.

### Construction of *amiSlARF5* vectors

amiRNAs were engineered into a 128 bp fragment containing the miRNA160a stem-loop, synthesized by Invitrogen, and then cloned into the *Bam*H I and *Hind* III sites of a modified pCAMBIA1301 vector with a 35 S promoter inserted using *Kpn* I and *Sma* I, which was used for plant transformation. The detailed protocol for generating amiRNAs is provided in Supplementary Data S1. The pCAMBIA1301 plasmid with a *35SCaMV* promoter was used as a control for plant transformation. Transgenic tomato plants were generated using the *amiSlARF5* vectors by the *Agrobacterium*-mediated leaf disk method, as described by Sun *et al*.^69^, with the exception that the antibiotics were replaced with 6 mg/L hygromycin and 300 mg/L Timentin.

For the detection of *amiSlARF5* lines to select positive transgenic plants, root materials of the transgenics were assayed for GUS activity^70^. To confirm that the T-DNA sequence of *ami*RNA *SlARF5* had been transformed into tomato plants, a region of 285 bp DNA from leaf materials was amplified by PCR detection (TaKaRa). The specific amiRNA fragment sense primer, (5-ATGAGACGGATTTCGTGTTT-3) was used in combination with a primer from the pCAMBIA1301 vector (5-CGACAGTGGTCCCAAAGAT-3) to amplify the DNA fragment. PCR procedures included an initial denaturation at 94 °C for 3 min, 30 cycles of 94 °C for 30 s, 55 °C for 30 s, and 72 °C for 1 min plus a final extension at 72 °C for 7 min.

### Microscopy

To estimate the cell area, cell numbers and cell layers in the ovaries and developing fruits of both wild-type and transgenic *SlARF5* plants, five fruits per line in each stage were sampled for analysis using fluorescence microscopy. Ovaries and young fruits were fixed with 2.5% glutaraldehyde overnight, rinsed with 0.1 M phosphate buffer three times, then transferred to 1% osmic acid for 1–2 h. The specimens were again washed with 0.1 M phosphate buffer and then dehydrated through a graded series of ethanol solutions. Samples were embedded in Spurr’s resin, and semithin sections were obtained and stained with 1% methylene blue and viewed using fluorescence microscopy (DMLB, Leica, Germany). The micrographs were captured with a Leica DFC 420 C camera using the Leica Application Suite software (Leica Microsystems, Germany).

### Quantitative real-time PCR

Total RNA was extracted from tomato ovaries using the TRIzol reagent (Invitrogen, Germany), according to the manufacturer’s instructions. The first cDNA strand was generated using the Improm-TM Reverse Transcription system (Promega, Madison, WI, USA), following the manufacturer’s protocol. The qPCR experiment was carried out using SYBR Premix Ex Taq (Takara) in a BioRad CFX96 Real-time PCR Detection System, as described by Wu *et al*.^38^, with three biological replications and three technical replications. The *Ubi3* gene (GenBank accession number X58253) was used as an internal control, and the relative expression was calculated using the comparative 2^(−*ΔΔ*Ct)^ method^71^. The primer pairs for qRT-PCR are listed in Supplementary Table S2.

### RNA sequencing and data analysis

Tissues were collected from pollinated fruits during the 3–4 mm diameter stage from amiRNA *SlARF5* lines and wild-type plants. Total RNA was extracted using TRIzol reagent (Invitrogen, CA, USA), according to the manufacturer’s instructions with two biological replicates. Four RNA libraries were sequenced using a Hiseq. 2000/2500 sequencing system (Illumina), following the manufacturer’s protocol.

Following the removal of low-quality reads, the sequenced RNA-seq reads were aligned to the tomato genome using Tophat version 2.0.8b with modified parameters^72^. The aligned read files were handled by Cufflinks, and the relative abundances of the transcripts were measured by the normalized RNA-seq fragment counts. Expression levels of each gene in FPKM (fragment per kilobase of exon per million fragments mapped) and the fold change of each gene were calculated. Comparisons with a q-value less than 0.05 were used for screening differentially expressed genes and further analyzed by enrichment of gene ontology (GO) terms and the Kyoto Encyclopedia of Genes and Genomes (KEGG) database. GO analysis was performed on differentially expressed genes using the Plant MetGenMAP database. Pathway enrichment analysis was performed using the KEGG database.

### Statistical Analysis

ANOVA statistical analyses were performed on all data using SPSS 15.0 (*p* < 0.01) and data were tested for significant differences (*p* < 0.05) using Tukey’s test. Means and standard errors were calculated from three biological replicates.

## Electronic supplementary material

Supplementary Information
