# Supplementary material for: Tomato AUXIN RESPONSE FACTOR 5 regulates fruit set and development via the mediation of auxin and gibberellin signaling
Source: Sci Rep. 2018 Feb 14;8:2971. doi: 10.1038/s41598-018-21315-y (PMC5813154; doi:10.1038/s41598-018-21315-y)
Supplement: Supplementary file 1 — Supplementary Information [file 41598_2018_21315_MOESM1_ESM.pdf]

Tomato AUXIN RESPONSE FACTOR 5 regulates fruit set and development via the mediation of auxin and gibberellin signaling

Songyu Liu[1], Youwei Zhang[1], Qiushuo Feng[1], Li Qin[1], Changtian Pan[1], Anthony Tumbeh Lamin-Samu[1], Gang Lu[1, 2]*

[1]Key Laboratory of Horticultural Plant Growth, Development and Quality Improvement, Ministry of Agricultural, Department of Horticulture, Zhejiang University, Hangzhou 310058, China

[2]Zhejiang Provincial Key Laboratory of Horticultural Plant Integrative Biology, Zhejiang University, Hangzhou 310058, China

*Corresponding author: Professor Gang Lu

E-mail: glu@zju.edu.cn, Tel: 86-571-88982277.

Address: 866 Yuhangtang Road, Hangzhou 310058, Zhejiang Province, China.

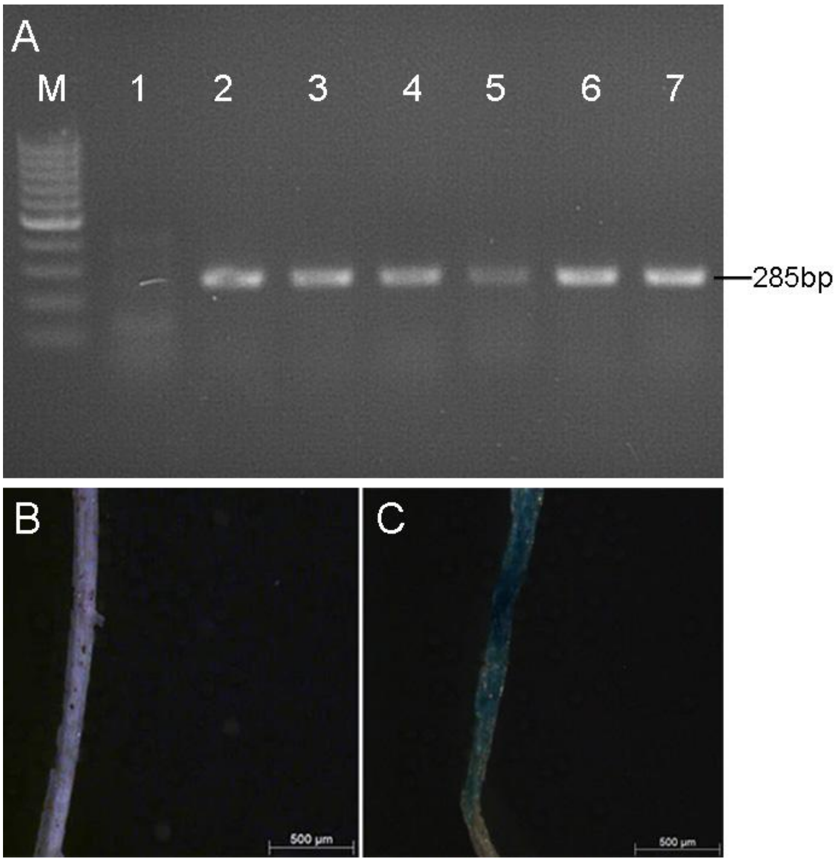

Figure S1. Detection of the transgenic tomato "Micro-Tom" plants with P35s:1301 *amiRNA ARF5*. (**A**) PCR analysis. M indicates DNA ladder; lane 1 is the negative control; lanes 2-6 are the *35S-amiARF5* transgenic tomato plants; lane 7 is the positive control. (**B-C**) GUS staining of wild-type tomato root tips (**B**), and transgenic plant root (**C**).

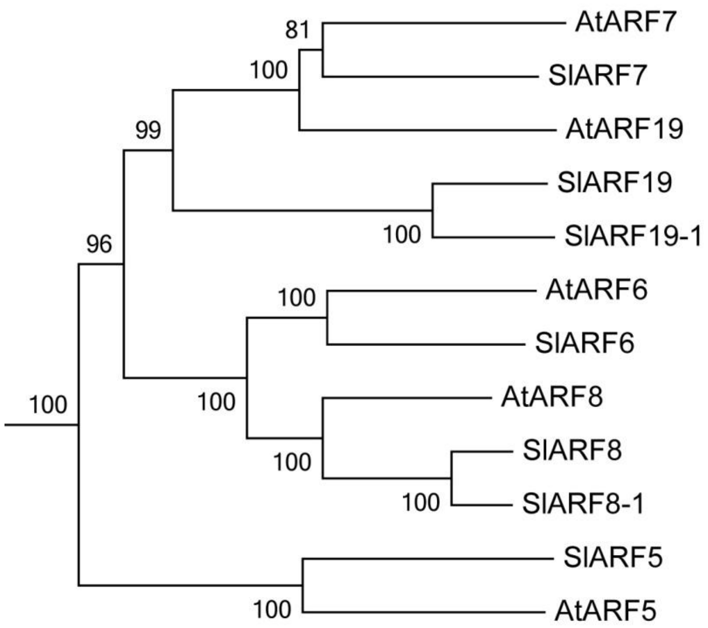

Figure S2. Neighbor-joining tree for the transcription of ARF5 proteins and their known homologs in tomato and Arabidopsis.

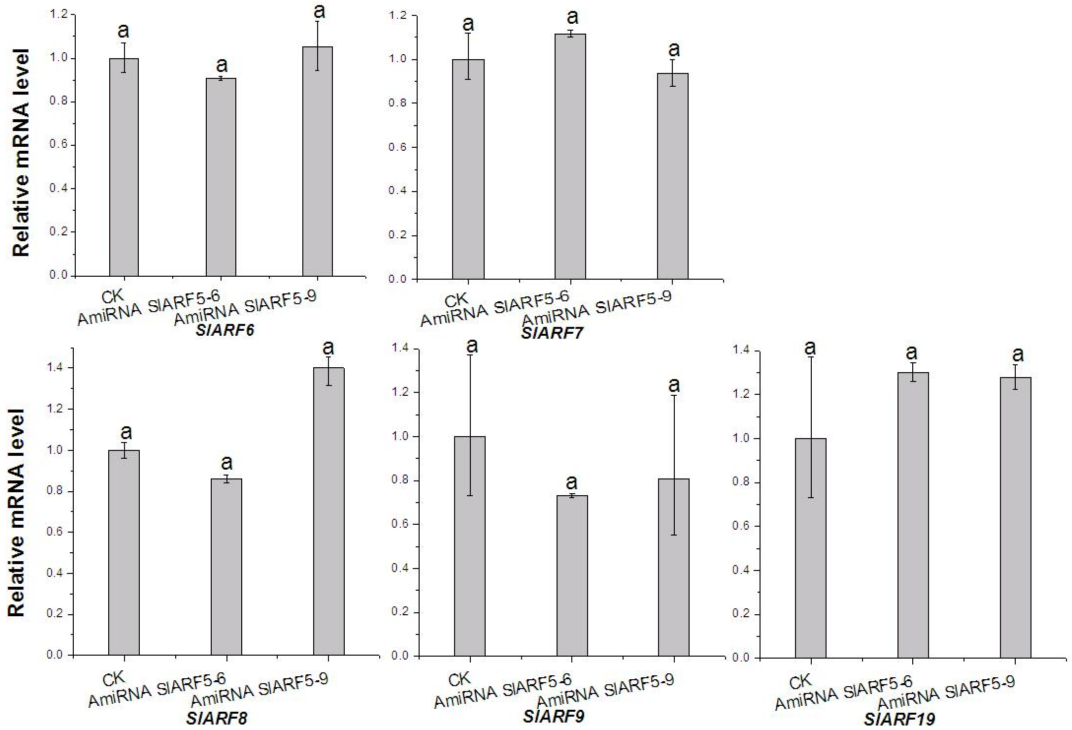

Figure S3. Expression analyses of *SlARF6*, *SlARF7*, *SlARF8*, *SlARF9* and *SlARF19* in wild type and *amiSlARF5* transgenic lines. CK: Wild type, *amiSlARF5-6* and *amiSlARF5-9*: transgenic plant lines. Flowers at anthesis (emasculated 2 days before) were collected. Standard errors are indicated with three replicates. ANOVA statistical analyses were performed with SPSS 15.0.

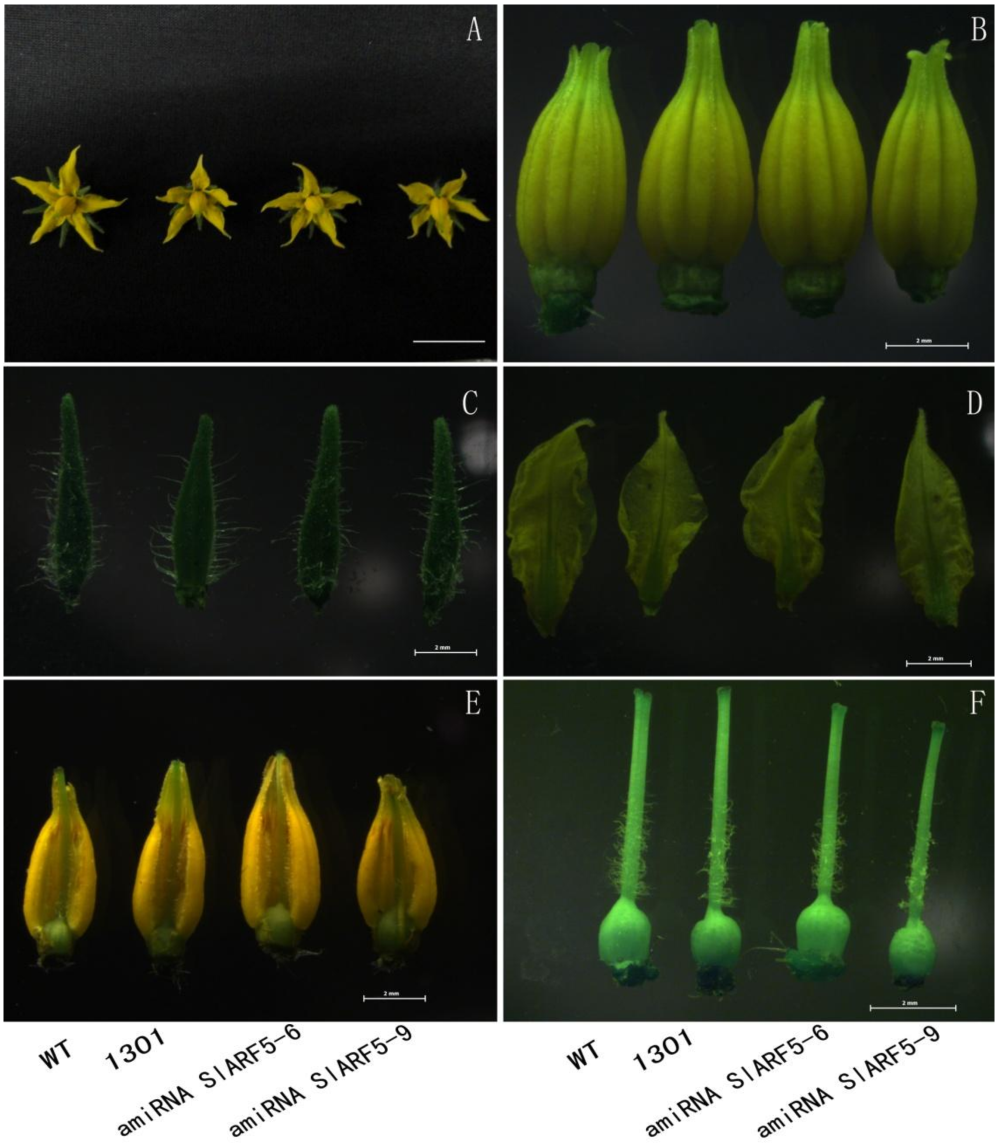

Figure S4. The plant and flower morphology of *amiSlARF5* transgenic plants. (A) Flower. **(B)** anther. **(C)** Sepal. **(D)** Petal. **(E)** Stamen and ovary. **(F)** Pistil. **(A-F)** WT, empty vector control, *amiSlARF5*-6 line, *amiSlARF5*-9 line are shown at each panel from left to right, respectively.

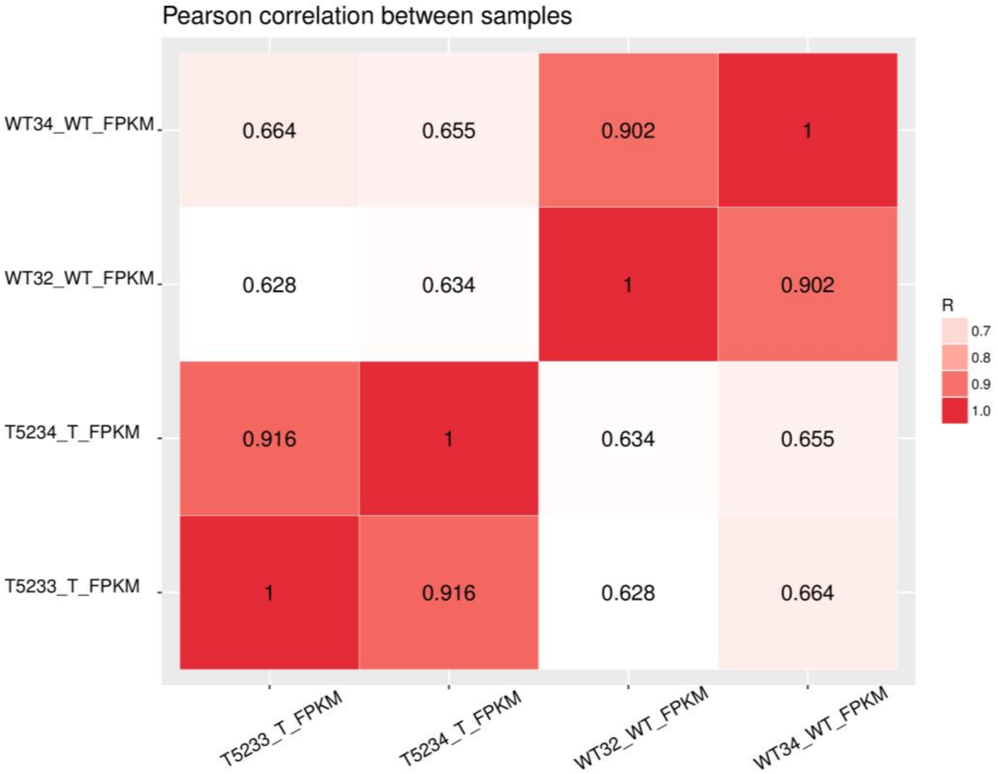

Figure S5. Correlation analysis (through Pearson's correlation coefficient) of RNA-seq data between two replicates.

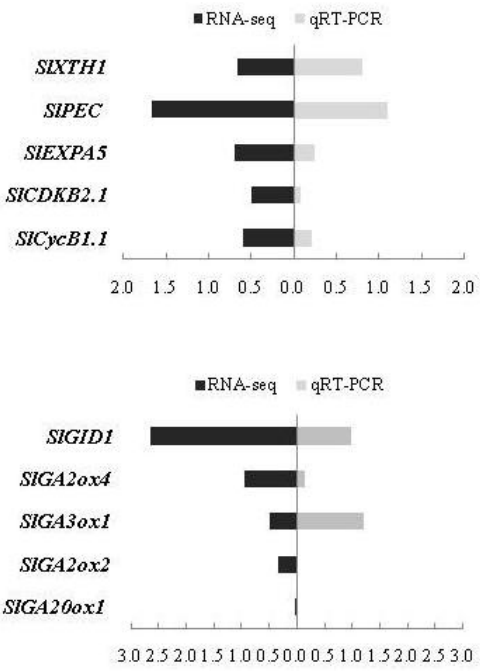

Figure S6. Comparison of qRT-PCR and RNA-Seq data on some differentially expression genes. The pollinated fruit tissues were collected in the 3–4 mm diameter stage from *ami*RNA *SlARF5* lines and wild-type plants. The fold change was calculat--ed by mRNA level multiple of transgenic relative to wild type.

Supplementary Table S1. The data set summary of transcriptome sequence.

Sample	Raw Data		Valid Data		Valid Ratio (reads)	Q20%	Q30%	GC content %
	Read	Base	Read	Base				
Ami5-1	45565334	4.56G	44777936	4.48G	98.27	98.6	92.11	45
Ami5-2	47296466	4.73G	46453930	4.65G	98.22	98.75	93.02	44
WT-1	53046632	5.30G	52116704	5.21G	98.25	99.34	95.3	42
WT-2	53606502	5.36G	52671138	5.27G	98.26	99.52	95.49	42

Supplementary Table S2. All gene-specific primers used for expression analysis by qRT-PCR.

Gene name	Forward (5’-3’)	Reverse (5’-3’)
Slubi3	AGAAGAAGACCTACACCAAGCC	TCCCAAGGGTTGTCACATACATC
SlARF5	ATTAGTTCTGAGTTGTGGC	GGTATCTGTGAAGTTGCTG
SlARF6	AGTGTCTCCTTCCTCATCATC	ATTGTTTTGGCTAACTGCTAC
SlARF7	AATTGGGTCGCTTAATAATATCCATT	TGCGACTGCTGCATGCA
SlARF8	TCGAATGCGGACGTTTACC	TAGCCTGAGTAACGTGCGATGT
SlARF9	CCAAGTTATCCTAATCTTCCTTCC	GTAAAGCCTCCTGGTCATATTTG
SlARF19	AATGGCTTCCGATTATGTCACC	TCCTACTGCACCACGCTTGTAC
SlARF12	GGAGGCATCAACAAATCAGG	CTTCGGGCAACAAAGCAATC
SlCDKB2.1	ATGCTGGTAAGAGTGTATCGG	CGGAGAGTAGTTGGAGGAAC
SlCycB1.1	CGTTACTAGGAGGTCTGCTG	CCTTTAGTTACAAGAGGCTTCG
SlPEC	ATGGGAAGGATCATGGAGACAGTGG	AAGGAAGAGGACTTCGCAGCTAAGC
SlXTH1	CTGCCACGCCACAAGAAGTCC	TTTGACGAACCCAACGAAGTCTCC
SlEXPA5	AAGGGTTCAAGAACTCAATGGCAAC	ACCATCGCCTGTAGTGACCTTAAAG
SlGH3-like	TGTGACATAGTCCCAGTAACAATAACATCG	TTGAAATGGAATGTAGTAAAGAGTCATGGAAAGG
SlGA20ox1	CTCATTTCTAATGCTCATCGT	TGCAGATGATTCTTTCTTAGCG
SlGA3ox1	GGCATTAGTAGTTAATATAGGTGA	AAATAAGCTACAGAAAGTCGATA
SlGA2ox2	ATTAAGATCCAATAACACTTCG	TCTTGATTTCACACTATTTGC
SlGA2ox4	ATGGAAGGAAAAGACAGTTTA	CTTTTCTCAAATAGGACCAAC
SlIAA1	GCTCTAATGATCCTGCTAAG	CACCGTCAACACTAACTTTC
SlIAA2	TATGAACCCACAACACCC	GGCTCCATCCATGCTAAC
SlIAA14	GGTTTACCAGGGAGGACAG	GGGATCTGAAATGGAGTTTG
SlGID1	GATCTTGATACACCTCTCAGTACTA	ACAGCCTTACATATACTAACAAGAC
SlGAST1	CAACAACAGAGAAATAACCAAC	TTATACGATGTCTTTGAACACC

Supplementary Data S1. A detailed protocol for construction of artificial miRNAs

The precursor of an endogenous *Arabidopsis thaliana* miRNA, mirRNA160a, as a backbone to express an artificial miRNA (amiRNA).

miR160a:

```
Structure 1 Folding bases 1 to 113 of 16Oct17-03-30-18
Initial ΔG = -49.90

        10        20        30        40        50
       AAU  C               CA   C         CCAA    C---|  AA
GGGUGAG   CU CAUGUUGGAGAAG  GGG ACGUGCAAA    CAAA    ACG  \
CCCACUU   GA GUACAACCUCUUC  CUC UGCACGUUU    GUUU    UGC  A
       CUC  -               AA   A         AUUC    ACUC^  CU
 110       100        90        80        70        60
```

AmiRNA sequence was designed using http://wmd3.weigelworld.org/cgi-bin/webapp.cgi?page=Homeproject=stdw md, which replace the miRNA and miRNA* sequences in the pre-miR160a with amiRNA and amiRNA* sequences.

amiRNA SlARF5:

```
Structure 1 Folding bases 1 to 113 of 16Oct17-03-32-18
Initial ΔG = -51.20

        10        20        30        40        50
       AAU  C               CA   U         CCAA    C---|  AA
GGGUGAG   CU CAUGUUUAUCAAU  CUG GCCCCCGAA    CAAA    ACG  \
CCCACUU   GA GUACAAAUAGUUA  GAC CGGGGGCUU    GUUU    UGC  A
       CUC  -               UC   C         AUUC    ACUC^  CU
 110       100        90        80        70        60
```

A 128 bp fragment synthesized by intron company containing amiRNA *SlARF5* was cloned into PMD18-T Simple vector .

Then the amiRNAs fragment from PMD18-T simple vector was cloned into *Bam*H I and *Hind* III sites of pCAMBIA1301-35S.
